# Nonselective Blocking of the Sympathetic Nervous System Decreases Detrusor Overactivity in Spontaneously Hypertensive Rats

**DOI:** 10.3390/ijms13045048

**Published:** 2012-04-23

**Authors:** Khae-Hawn Kim, Long-Hu Jin, Gwoan-Youb Choo, Hun-Jae Lee, Bo-Hwa Choi, Jiyeon Kwak, Sang-Min Yoon, Chang-Shin Park, Tack Lee

**Affiliations:** 1 Departments of Urology, Gachon University Gil Hospital, Incheon 405-760, Korea; E-Mail: kimcho99@gilhospital.com; 2 Departments of Urology, Inha University College of Medicine, Incheon 402-751, Korea; E-Mails: jinlonghu_2021@hotmail.com (L.-H.J.); mr_uro@naver.com (G.-Y.C.); uroyoon@inha.ac.kr (S.-M.Y.); 3 Department of Preventive and Social Medicine, Inha University College of Medicine, Incheon 402-751, Korea; E-Mail: lee4146@inha.ac.kr; 4 Department of Pharmacology, Inha University College of Medicine, Incheon 402-751, Korea; E-Mail: bo813@nate.com; 5 Department of Physiology and Biophysics, Inha university College of Medicine, Incheon 402-751, Korea; E-Mail: kwak1014@inha.ac.kr

**Keywords:** autonomic nervous system, detrusor overactivity, labetalol

## Abstract

The involuntary dual control systems of the autonomic nervous system (ANS) in the bladder of awake spontaneously hypertensive rats (SHRs) were investigated through simultaneous registrations of intravesical and intraabdominal pressures to observe detrusor overactivity (DO) objectively as a core symptom of an overactive bladder. SHRs (*n* = 6) showed the features of overactive bladder syndrome during urodynamic study, especially DO during the filling phase. After injection of the nonselective sympathetic blocking agent labetalol, DO disappeared in 3 of 6 SHRs (50%). DO frequency decreased from 0.98 ± 0.22 min^−1^ to 0.28 ± 0.19 min^−1^ (*p* < 0.01), and DO pressure decreased from 3.82 ± 0.57 cm H_2_O to 1.90 ± 0.86 cm H_2_O (*p* < 0.05). This suggests that the DO originating from the overactive parasympathetic nervous system is attenuated by the nonselective blocking of the sympathetic nervous system. The detailed mechanism behind this result is still not known, but parasympathetic overactivity seems to require overactive sympathetic nervous system activity in a kind of balance between these two systems. These findings are consistent with recent clinical findings suggesting that patients with idiopathic overactive bladder may have ANS dysfunction, particularly a sympathetic dysfunction. The search for newer and better drugs than the current anticholinergic drugs as the mainstay for overactive bladder will be fueled by our research on these sympathetic mechanisms. Further studies of this principle are required.

## 1. Introduction

Overactive bladder (OAB) is a bothersome medical condition that is defined by the core symptom of urinary urgency [1]. Urodynamic studies in humans have shown that this urinary urgency results from sudden, involuntary detrusor contraction during the filling phase of urination [2]. Overactive bladder is a highly prevalent health problem worldwide with an incidence that ranges from 16% to 17% in the general population [3]. The age-specific prevalence of OAB is similar among men and women [4]. Overactive bladder has as meaningful negative impacts on health-related quality of life as other chronic diseases, such as diabetes and hypertension, do [5]. Although recent advances in our understanding of OAB pathophysiology have led to improved treatment options, the current drug therapy is still far from optimal because many aspects of the pathophysiology of OAB are not yet completely understood.

The normal functions of the bladder are mainly under the involuntary control of the autonomic nervous system (ANS), although this involuntary control is mixed with some voluntary control of somatic nerves. The involuntary control derives from a dual set of control systems of sympathetic and parasympathetic nerves that generally work in opposition in their effect on target organs [6]. In the bladder, the neural control depends on appropriate changes in the balance between the sympathetic and the parasympathetic nervous systems through the filling and voiding phases [7]. Recently, trials in humans have proved that autonomic dysfunction is involved in the pathogenesis of OAB [8–11]. Because the parasympathetic nervous system is responsible for the detrusor contraction, the detrusor overactivity (DO) in OAB represents overactive parasympathetic activity. However, it has remained unknown whether sympathetic activity, the opposing partner in bladder function, is increased or decreased during DO in patients with OAB.

Thus, the present study was undertaken to compare bladder function and some characteristics of DO during the filling phase before and after the administration of the nonselective sympathetic blocking agent labetalol. For this purpose, the intraabdominal and intravesical pressures were measured simultaneously in spontaneously hypertensive rats (SHRs), which are a model system for studying DO and altered sympathetic activity. The study also aimed to test the hypothesis that the dual-control systems of the ANS are closely related and that upregulated sympathetic activity is required to maintain the parasympathetic overactivity representative of DO in SHRs.

## 2. Results and Discussion

### 2.1. Results

#### 2.1.1. Body and Bladder Weights, Normalized Ratios between Them, and Systemic Blood Pressure

**Figure 1:**
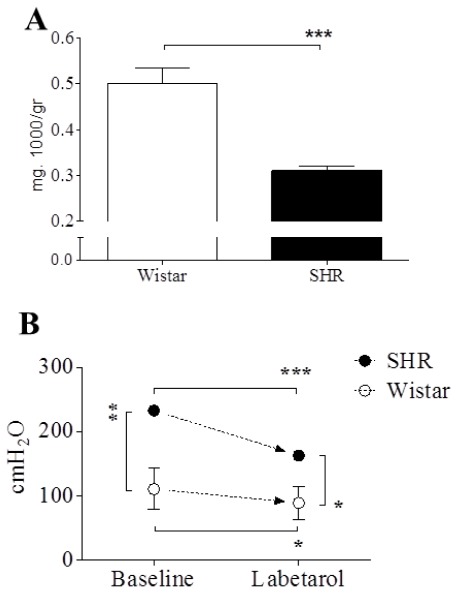
Ratio of bladder weight to body weight (**A**) and effects of intraarterial labetalol (5 mg·kg^−1^) on mean arterial blood pressure (**B**) in conscious Wistar and spontaneously hypertensive rats (SHRs). The ratio of bladder weight to body weight was significantly lower in SHRs than in Wistar rats. The mean arterial pressures (time-averaged arterial pressure over 10 min) of SHRs were significantly higher than those of Wistar rats (*p <* 0.01). After labetalol injection, mean arterial pressures decreased in both rats. Results are expressed as mean ± standard error of the mean. * *p* < 0.05, ** *p* < 0.01, *** *p* < 0.001 (unpaired Student’s *t*-test).

SHRs were significantly lighter than Wistar rats (348.30 ± 7.03 and 390.00 ± 4.28 g, respectively). The bladder weights of SHRs were also significantly less than those of Wistar rats (1.08 ± 0.04 and 1.96 ± 0.11 mg, respectively). When bladder weight (mg) was normalized to body weight (g), the ratio was significantly lower by 38.0% in SHRs than in Wistar rats (Figure 1A).

The mean arterial pressure (time-averaged arterial pressure over 10 min) was significantly different between Wistar rats (111.0 ± 32.0 SE cm H_2_O) and SHRs (233.0 ± 4.7 SE cm H_2_O) (*p <* 0.01). After the injection of labetalol, mean arterial pressures decreased to 89.4 ± 25.6 SE cm H_2_O (*p <* 0.001) in Wistar rats and 163.1 ± 4.7 SE cm H_2_O (*p <* 0.05) in SHRs, respectively. Even after labetalol injection, mean arterial pressure differed significantly between the groups (*p* < 0.05) (Figure 1B).

#### 2.1.2. Cystometric Parameters before and after the Intraarterial Injection of Labetalol

**Figure 2:**
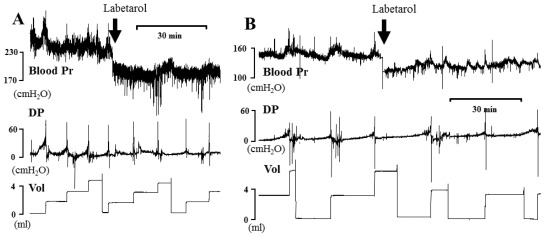
Representative cystometrogram showing simultaneous arterial pressures, detrusor pressures, and micturition volume before and after the arterial injection of 5 mg·kg^−1^ labetalol in conscious SHRs (**A**) and Wistar rats (**B**). Pr: pressure; DP: detrusor pressure; Vol: volume.

**Figure 3:**
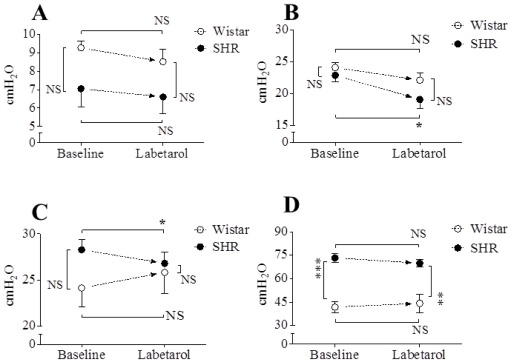
The effects of intraarterial 5 mg·kg^−1^ labetalol on the pressure parameters in SHRs and Wistar rats. (**A**) Basal pressure by detrusor pressure (DP); (**B**) Flow pressure; (**C**) Threshold pressure by DP; (**D**) Micturition pressure by DP. Results are expressed as mean ± standard error of the mean. * *p* < 0.05, ** *p* < 0.01 (unpaired Student’s *t*-test). NS: not significant.

**Figure 4:**
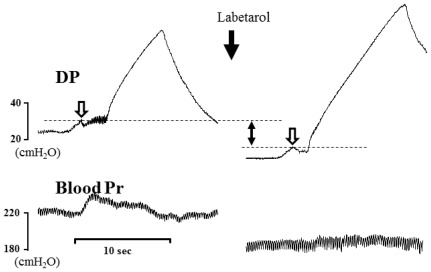
Representative cystometrogram showing simultaneous arterial pressures and flow pressure by detrusor pressure before and after the arterial injection of 5 mg·kg^−1^ labetalol in conscious SHRs. DP: detrusor pressure; Pr: pressure.

**Figure 5:**
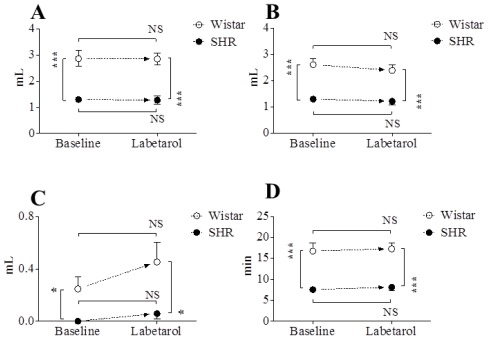
The effects of intraarterial 5 mg·kg^−1^ labetalol on the volume parameters in SHRs and Wistar rats. (**A**) Bladder capacity; (**B**) Micturition volume; (**C**) Residual volume; (**D**) Micturition interval. Results are expressed as mean ± standard error of the mean. * *p* < 0.05, ** *p* < 0.01, *** *p* < 0.001 (unpaired Student’s *t*-test). NS: not significant.

**Figure 6:**
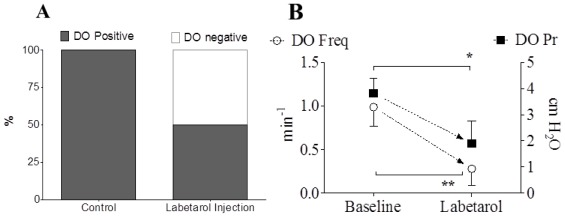
The effects of intraarterial 5 mg·kg^−1^ labetalol on the cystometric detrusor overactivity (DO)-related parameters during the filling phase in SHRs and Wistar rats. (**A**) DO occurrence rate; (**B**) DO frequency and pressure. Before the injection of labetalol, DO proved by IAP was shown in all SHRs. After the injection of labetalol, DO disappeared in 3 of 6 SHRs. Also, DO frequency and pressure decreased significantly. Results are expressed as mean ± standard error of the mean. * *p* < 0.05, ** *p* < 0.01 (unpaired Student’s *t*-test).

Representative cystometric tracings from Wistar rats and SHRs before and after the injection of labetalol are shown in Figure 2. All curves of the micturition cycles with arterial blood pressures were reproducibly recorded in all animals, and the comparisons of all pressure parameters were performed on the basis of detrusor pressure, which was defined as the difference between IVP and IAP. Before the injection of labetalol, there were no significant differences in BP, FP, or TP between Wistar rats and SHRs (Figure 3A–C). However, MP was significantly higher in SHRs than in Wistar rats (Figure 3D). After intraarterial injection of labetalol, the SHRs showed decreased values in only FP (*p* < 0.05) and TP (*p* < 0.05), whereas the Wistar rats showed no changes in any of the pressure parameters (Figures 3,4). After the injection of labetalol, there were no significant differences between the Wistar rats and the SHRs in any pressure parameters except MP. All volume parameters, including BC (*p* < 0.001), MV (*p* < 0.001), RV (*p* < 0.05), and MI (*p* < 0.001), were lower in SHRs than in Wistar rats before the injection of labetalol. Labetalol injection did not change any volume parameters in either group. After labetalol injection, all volume parameters were still significantly lower in SHRs than in Wistar rats (Figure 5). Before the injection of labetalol, DO proved by IAP was not shown in Wistar rats, but it was shown in all SHRs. After the injection of labetalol, DO disappeared in 3 of 6 SHRs (50%) (Figure 6A). In addition, DO frequency decreased from 0.98 ± 0.22 min^−1^ to 0.28 ± 0.19 min^−1^ (*p* < 0.01), and DO pressure decreased from 3.82 ± 0.57 cm H_2_O to 1.90 ± 0.86 cm H_2_O (*p* < 0.05) (Figure 6B).

### 2.2. Discussion

The results of the present study showed that intraarterial labetalol, a nonselective sympathetic blocking agent, objectively decreased the occurrence, frequency, and pressure of DO in SHRs with a lowering of systemic blood pressure. This suggests that the DO originating from the overactive parasympathetic nervous system is attenuated by the nonselective blocking of the overactive sympathetic nervous system, which is a major characteristic of SHRs. The detailed mechanism of this result remains unknown, but parasympathetic overactivity seems to require overactive sympathetic nervous system activity in a kind of balance between the two systems. Furthermore, this finding is consistent with the recent clinical findings that patients with idiopathic OAB may have ANS dysfunction, particularly sympathetic dysfunction.

The human nervous system is a network within which the ANS plays an important role in controlling the many vital functions of visceral organs, such as heart rate, respiration rate, digestion, and micturition, both automatically and involuntarily. This innervation is divided into the sympathetic and the parasympathetic nervous systems, which usually work in opposition to each other [7]. Anatomically, each of the systems has a different origin located between the central nervous system and the target organs, such as from the thoracic and lumbar spinal cord in the sympathetic nervous system and from the craniosacral spinal cord in the parasympathetic nervous system. The two systems also have a unique sequential two-neuron efferent pathway, in which the preganglionic neuron synapses onto a postganglionic neuron before reaching the target organ, but the innervations vary according to the functions of the target organs [6,12]. Thus, the anatomical arrangements of the ANS are so complex, nuanced, and unique that it is impossible to draw any grounded conclusions about the normal functions of the ANS and how much “normal” is changed in various disease states.

Despite the expected important role of the ANS in OAB, few studies have examined the exact role of the ANS in the pathophysiology of OAB because of difficulties in methodology. However, growing evidence suggests that an imbalance between the sympathetic and parasympathetic activity of the ANS is involved in the pathophysiology of OAB [8,9,11]. Some epidemiologic studies in men with symptomatic benign prostatic hyperplasia have suggested that those symptoms may be closely related to sympathetic overactivity. For example, McVary *et al*. showed that validated measures of hyperactive ANS activity are significantly related to the most commonly used symptom scores in men with benign prostatic hyperplasia [13]. Kim *et al*. proved that female incontinent patients with urodynamic DO have sympathetic overactivity compared with parasympathetic activity as assessed through a frequency domain analysis on a heart rate variability test [11].

Previous studies using animal models such as SHRs also support a link between ANS hyperactivity and urodynamic voiding parameters [14,15], which agrees with our current results. SHRs, which were previously proved to have an overabundance of sympathetic nerve fibers innervating the bladder [16], void more frequently and have a smaller bladder capacity and higher maximal pressure with higher blood pressures during voiding than do control rats. Flow pressure, which was proved in our previous study [17], represents the urethral resistance to voiding, because the pressure is at the time of the start of flow of urine. In the current study, this pressure decreased as a result of the decreased internal sphincter activity after the global blocking of sympathetic activity.

Our ultimate success in controlling DO is still hampered by our incomplete knowledge of the mechanisms of DO. Most previous studies have focused on etiological and pathophysiological mechanisms inside the bladder, such as muscarinic receptors or gap junctions [5,6], and few studies have explored mechanisms beyond the bladder, especially the ANS. Theoretically, parasympathetic stimulation through the pelvic nerve induces detrusor contraction, whereas sympathetic stimulation through the hypogastric nerve induces detrusor relaxation and internal sphincter contraction. Normally during the storage phase, the parasympathetic signals to the bladder are suppressed by tonic inhibitory systems in the brain and the sympathetic pathways are activated by the spinal reflex [12]. However, patients with idiopathic OAB show increased parasympathetic activity during the filling phase [18]. Normally, both components of the ANS are in constant balance. If one is stronger, the other becomes stronger to compensate. Thus, the sympathetic activity in OAB may be stronger than that in the normal state.

In the current study, SHRs showed DO and high blood pressure. After global blocking of sympathetic activity, both the frequency and the pressure of DO were decreased with a lowering of blood pressure. Thus, the parasympathetic activity inducing DO via the spinal pathway seems to require the backup of sympathetic activity. Further studies are warranted to define the underlying mechanisms.

## 3. Experimental Section

### 3.1. Animals

Age-matched (14-week-old) 6 male SHRs and normotensive control 6 female Wistar/ST (Wistar) rats (Japan SLC, Hammatsu, Japan) weighing 200 to 250 g were used in this study. They were reared in our laboratory under controlled conditions with 12 h of light and 12 h of darkness and were allowed free access to standard food pellets and tap water. All experimental animal procedures were performed in accordance with the *Guide for the Care and Use of Laboratory Animals* of the National Institutes of Health and were approved by the local animal ethics committee.

### 3.2. Surgical Procedures

Implantations of the catheters for the recordings of intravesical pressure (IVP) and intraabdominal pressure (IAP) and drug injection were performed as described previously [19]. In brief, the rats were anaesthetized with ketamine (75 mg/kg intraperitoneally; Ketamine, Yuhan, Seoul, Korea) and xylazine (15 mg/kg intraperitoneally; Rompun, Bayer Korea Ltd, Seoul, Korea). Through a lower midline incision, a polyethylene catheter (PE-50; Becton Dickinson, Parsippany, NJ, USA) with a cuff was inserted into the dome of the bladder. To record IAP, a balloon (Latex, Dawoo Medical, Incheon, Korea) around the cuff of a catheter tip was placed proximal to the bladder and was tied to the other catheter to the bladder with silk tie. To record systemic arterial pressure, a catheter (PE-10; Becton Dickinson) was inserted into the lower abdominal aorta via the femoral artery. The catheters were tunneled subcutaneously and exited through the skin at the back of the animal. The free ends of the catheters were sealed. After surgery, each rat was caged individually and maintained in the same manner.

### 3.3. *In Vivo* Cystometric Investigations and Analysis

Three days after the catheter implantations, mean arterial pressures were measured for about 10 min before and after the injection of labetalol in animals that were allowed to roam freely in the cage [20,21]. Labetalol (labetalol hydrochloride; 5 mg·kg^−1^) was injected through the catheter into the lower abdominal aorta. Before the cystometry, body weight was recorded. Cystometrograms were then obtained in unanaesthetized, unrestrained rats in metabolic cages. The indwelling catheter to the bladder was connected to a two-way valve that was connected via a T-tube to a pressure transducer (Research Grade Blood Pressure Transducer, Harvard Apparatus, Holliston, MA, USA) and a microinjection pump (PHD22/2000 pump, Harvard Apparatus). The other indwelling catheters, which were connected to an air-free abdominal balloon and the femoral artery, were connected to another pressure transducer to record the IAP. The balloon functions best when it is filled and stabilized to around 50 to 100 cm H_2_O of IAP. To prevent IAP and blood pressure decay by minute leakages through the multiple joints of the lines and connectors, Parafilm M laboratory sealing film (American National Can, Chicago, IL, USA) was wrapped around the joints. Micturition volumes were recorded by means of a fluid collector connected to a force-displacement transducer (Research Grade Isometric Transducer). Room-temperature saline was infused into the bladder continuously at a rate of 10 mL·h^−1^. IVP, IAP, and micturition volumes were recorded continuously with Acq Knowledge 3.8.1 software and an MP150 data acquisition system (Biopac Systems Inc., Goleta, CA, USA) at a sampling rate of 100 Hz. The mean values from three reproducible micturition cycles were used for evaluation. IAP was defined as the recorded balloon pressure corrected by subtracting the lowest balloon pressure in each voiding cycle, which is comparable to zeroing in human cystometry. Detrusor pressure was defined as IVP minus IAP. The IVP rises during the filling phase were defined as increments of IVP that exceeded 2 cm H_2_O from baseline, which was interpreted as abdominal straining if occurring with simultaneous similar changes in IAP, or as DO if occurring without simultaneous similar changes in IAP [22]. Flow pressure (FP) was defined as the IVP or detrusor pressure at the time of the start of flow of urine from the urethra, which was detected by applying a small electric current to a circuit grid beneath the rat [17].

The following two kinds of cystometric parameters were investigated:

- ***Pressure- and volume-related parameters derived from detrusor pressure***: Basal pressure (BP; the lowest bladder pressure during filling), FP (bladder pressure at the time of start of flow of urine from the urethra), threshold pressure (TP; bladder pressure immediately before micturition), maximum pressure (MP; maximum bladder pressure during the micturition cycle), micturition volume (MV; volume of expelled urine), residual volume (RV; remaining urine after voiding), bladder capacity (BC; MV + RV), and micturition interval (MI; intervals between micturition contractions).
- ***DO-related parameters during the filling phase***: Time of filling phase (interval from the initiation of infusion through the tube and the point immediately before the initiation of micturition), frequency of abdominal straining per minute, frequency of DO per minute, and increased amplitude from base to peak of DO spike as IVP. These frequencies were calculated on the basis of the time of filling phase.

After cystometry, the animals were sacrificed by cervical dislocation. The bladder and urethra were removed *en bloc* and separated at the level of the bladder neck and the bladder was weighed.

### 3.4. Statistical Analyses

The results are expressed as mean values ± standard errors of the mean. Normal distributions were confirmed by the Shapiro-Wilks’ W test. Statistical analyses were undertaken with unpaired Student’s *t-*tests or a one-way analysis of variance with the Tukey post-hoc test for multiple comparisons. The data for DO incidence were expressed as percentages of animals showing DO. All analyses were performed with GraphPad Prism, version 5.03, 2009 (GraphPad Software Inc., La Jolla, CA, USA). *p* < 0.05 was considered statistically significant.

## 4. Conclusions

We demonstrated herein that intraarterial labetalol, a nonselective sympathetic blocking agent, suppresses the occurrence, frequency, and pressure of DO in SHRs. This suggests that the DO from the overactive parasympathetic nervous system requires compensatory overactive sympathetic nervous system activity in a kind of balance between these two systems. The current mainstay of pharmacological therapies for OAB is still anticholinergic drugs [23], which suppress the overactive parasympathetic nervous system during the filling phase. These competitive inhibitors of acetylcholine have no significant effect on voiding because there is a massive release of acetylcholine during voiding compared with during the storage phase. However, these drugs can produce voiding difficulty or urinary retention in clinical practice, especially in patients with benign prostatic hyperplasia. The search for newer and better drugs will be fueled by our research on the mechanisms of sympathetic nervous system activity. Further studies of this principle are required.
